# Robotic-Assisted Surgery in Emergency General Surgery: A Prospective, Single-Center, Case Series

**DOI:** 10.7759/cureus.93650

**Published:** 2025-10-01

**Authors:** Thalia Petropoulou, Kyriacos Evangelou, Andreas Polydorou

**Affiliations:** 1 Department of General Surgery, Brighton and Sussex University Hospitals NHS Trust, Brighton, GBR; 2 Department of Robotic Colorectal Surgery, Euroclinic General Hospital, Athens, GRC; 3 Department of General Surgery, Aretaieion University Hospital, Athens, GRC; 4 Department of Medicine, National and Kapodistrian University of Athens, Athens, GRC

**Keywords:** emergency robotic surgery, emergency surgery, minimally invasive surgery, robotic-assisted surgery, surgical expertise

## Abstract

Introduction: Robotic-assisted surgery has transformed elective general surgery, offering well-documented benefits for both surgeons and patients. However, its application in emergency settings remains underexplored. This study aimed to evaluate the feasibility, safety, and perioperative outcomes of robotic surgery in emergency general surgical cases when performed by an experienced surgical team.

Methods: This observational, single-center study included 12 patients who underwent emergency robotic surgery at a high-volume tertiary care institution. All procedures were performed by the same surgeon with extensive experience in minimally invasive and robotic techniques. Demographic data and perioperative outcomes were prospectively recorded. Given the small sample size and single-arm design, only descriptive statistics were reported. Continuous variables are presented as mean ± standard deviation (SD) and median (range), and categorical variables as counts and percentages. No inferential statistical testing was applied, as the study was not powered for comparative analysis.

Results: The median patient age was 73 years (range 38-91), with a median BMI of 27 kg/m² (range 25-34). The most common indications were obstructing colon tumors (58.3%) and incarcerated hernias (16.7%). The median operative time was three hours (range, two to six hours). There were no conversions to open or laparoscopic surgery. Minor postoperative complications (Clavien-Dindo grade I-II) occurred in two patients (16.7%) and were managed conservatively. One intraoperative complication (ureteric injury) was identified and managed robotically without conversion. No patient required postoperative intensive care. The median time to first flatus and oral intake was two days (range, one to three days). The median hospital stay was two days (range, 1-11 days), with no 90-day readmissions or mortality.

Conclusion: Robotic-assisted emergency general surgery may be a safe and feasible option in carefully selected patients when performed by an experienced surgical team with institutional support. While early results from this small cohort are favorable, further multicenter comparative studies are needed to validate these findings and define optimal patient selection.

## Introduction

Robotic-assisted surgery has become increasingly established for elective general and colorectal procedures, ranging from routine cholecystectomy to complex multisegment colectomy for malignancy [1,2]. Its well-documented advantages include enhanced three-dimensional (3D) visualization, tremor filtration, improved dexterity through articulated instruments, and superior surgeon ergonomics [3]. However, its role in emergency general surgery, where time pressure, unpredictable anatomy, and complex physiology converge, remains underexplored.

Emergency surgery carries significantly higher morbidity and mortality than elective procedures, with global mortality rates of 5-15% and complication rates exceeding 30% in high-risk populations [4,5]. Patients often present with acute, life-threatening conditions, such as perforation, obstruction, strangulated hernia, or trauma, requiring rapid intervention, frequently before full preoperative optimization or comprehensive imaging can be performed. These challenges are compounded by comorbidities, altered anatomy from prior surgeries, and intraoperative findings such as dense adhesions, bowel edema, or active bleeding [6].

Open surgery remains the predominant approach in emergencies due to its speed and universal availability [7]; however, it is associated with greater tissue trauma, longer recovery, and higher rates of surgical site infection [8]. Laparoscopy offers minimally invasive benefits, reduced postoperative pain, earlier mobilization, and shorter hospital stays, but its performance in emergencies is limited by reduced instrument maneuverability, two-dimensional (2D) visualization in some systems, and difficulty managing massively distended bowel or deep pelvic pathology [9,10]. Set-up challenges in unstable patients and the risk of conversion also remain concerns [11].

Robotic-assisted surgery has the potential to address several of these limitations. Compared with laparoscopy, robotic systems provide stable 3D magnification, greater degrees of instrument articulation, tremor elimination, and improved ergonomics, which may enhance precision in adhesiolysis, deep pelvic dissection, and complex suturing under urgent conditions [12,13]. While concerns persist regarding set-up time in emergencies, studies suggest that, in experienced hands, docking times can be kept under 10 minutes, comparable to laparoscopy [14,15]. Furthermore, although the risk of conversion is a major downside of robotic emergency surgeries, it remains significantly lower compared to laparoscopy [15].

Existing literature on robotic emergency general surgery is scarce, limited to small retrospective series and case reports, often focused on specific pathologies such as incarcerated hernia repair or colectomy for obstruction [12,16]. No consensus currently exists on optimal patient selection, and comparative outcomes versus laparoscopic and open approaches remain inadequately defined.

Given these gaps, we conducted a prospective single-center case series to evaluate the feasibility, safety, and clinical outcomes of robotic-assisted surgery for urgent general surgical indications, including obstructing colorectal tumors, incarcerated hernias, and other acute abdominal pathologies. All cases were performed by a high-volume robotic colorectal and general surgeon (>250 robotic procedures) supported by a dedicated multidisciplinary team trained in emergency robotic protocols. To our knowledge, this is among the first prospective clinical series to assess the real-world performance of robotic-assisted surgery in unselected emergency presentations meeting standard safety criteria, aiming to inform both feasibility and future research directions.

This article was previously published as a preprint on the open-access multidisciplinary platform, preprints.org, on August 11, 2025 [17]. 

## Materials and methods

Study design and ethical compliance

This was a prospective, observational, single-center study conducted at a high-volume tertiary care hospital between January 2020 and December 2024. The study adhered to the STROBE (Strengthening the Reporting of Observational Studies in Epidemiology) guidelines [18]. Ethical approval was obtained from the Institutional Review Board of Euroclinic, Athens (IRB No. 402022, dated April 14, 2022), and written informed consent was obtained from all participants.

Eligibility criteria

Inclusion Criteria

All adult patients (≥18 years) undergoing robotic-assisted surgery for urgent or emergent general surgical indications, including but not limited to obstructing colorectal malignancy, incarcerated or strangulated hernia, acute complicated diverticulitis with obstruction or localized perforation, and iatrogenic intraoperative injury repair during emergency procedures, were consecutively enrolled without time-of-day restrictions.

We defined “emergencies” as clinical scenarios necessitating urgent surgical intervention within 24 hours of diagnosis or emergency room (ER) presentation, due to acute pathology posing a risk of rapid deterioration if not promptly addressed. Examples include mechanical large bowel obstruction, incarcerated or strangulated hernia, acute diverticulitis with impending perforation or obstruction, and intraoperative iatrogenic injuries (such as ureteric injuries, usually manifesting as copious drainage from surgical drains, oliguria, or flank pain) requiring immediate repair. These cases were considered unsuitable for deferral to elective scheduling and required acute surgical management during the same hospital admission on an unplanned basis [19,20].

Exclusion Criteria

Patients with documented hemodynamic instability unresponsive to initial resuscitation, generalized peritonitis with septic shock requiring damage-control surgery, and/or any absolute contraindication to pneumoperitoneum (e.g. severe cardiopulmonary instability) were excluded from the study.

As the robotic system was accessible 24/7 during the study period, no cases were excluded due to lack of availability.

Sample size rationale

Given the exploratory nature of the primary objective, assessing the real-world feasibility and safety of robotic-assisted emergency general surgery, no formal a priori power calculation was performed. Instead, all eligible patients to be operated on during the predefined study period were consecutively enrolled. This approach is consistent with early-phase feasibility studies, which primarily aim to generate initial outcome data, evaluate workflow logistics, and identify potential safety signals, rather than to formally test hypotheses or detect statistically significant differences.

Surgical team and institutional experience

All procedures were performed by a board-certified consultant colorectal and general surgeon with >250 completed robotic procedures and formal fellowship training in robotic colorectal surgery. The surgeon was supported by two dedicated bedside assistants with >150 robotic cases each, a scrub nurse trained in robotic instrumentation, and an anesthesiologist experienced in prolonged minimally invasive procedures. The institution had performed >150 robotic general surgical cases before the study period, including complex oncologic resections.

The decision to perform robotic-assisted surgery in emergency cases was made on a case-by-case basis by the operating surgeon in consultation with the on-call multidisciplinary team. Robotic surgery was considered for patients who were hemodynamically stable, had pathology amenable to minimally invasive management, and had no contraindications to pneumoperitoneum. A trained team and 24/7 access to the robotic system ensured that robotic intervention could be delivered without delaying care. Patients requiring immediate open or damage-control surgery were excluded. This pragmatic, individualized approach reflects real-world clinical practice during the early adoption of robotics in emergency general surgery.

Surgical technique

Patients were positioned according to the target anatomy (modified Lloyd-Davis for colorectal cases, supine for hernia repair). In large bowel obstruction, intraoperative decompression was achieved prior to docking by one or more of the following: gentle manual decompression via small enterotomy (subsequently closed robotically), insertion of a transanal rectal tube for distal obstructions, or careful controlled trocar entry under vision to avoid injury to distended loops. Robotic port placement followed a standardized four-arm configuration for colectomies, with a 12-mm assistant port for suction, stapling, and specimen retrieval. In hernia repairs, a transabdominal preperitoneal (TAPP) approach was used.

Study variables

Demographic variables included sex, age, body mass index (BMI), prior abdominal surgery, and ASA (American Society of Anesthesiologists) score. Operative variables included indication for surgery, procedure type, operative time (OT) from skin incision to skin closure, drain placement, and estimated blood loss (EBL). Outcomes were classified as primary, which included conversion to open or laparoscopic surgery, and secondary, which included intraoperative complications, postoperative complications (Clavien-Dindo [21]), intensive care unit (ICU) admission, time to first flatus, mobilization, and oral intake, length of stay (LOS), 90-day readmission, and 90-day mortality.

Data sources and measurement

Data were prospectively recorded in electronic patient records. Complications were identified through daily inpatient review and follow-up at one, three, and 12 months for benign disease and at three-month intervals for oncologic cases.

Bias minimization

Consecutive patient inclusion minimized selection bias. All operations were performed by the same surgical team under consistent institutional protocols, limiting variability. Prospective data collection reduced recall bias, and all outcomes were defined according to standardized, validated classifications. Continuous 24/7 robotic system availability during the study period further reduced potential selection bias related to platform access.

Statistical analysis

Continuous variables are presented as mean ± standard deviation (SD) and median (range). Categorical variables are presented as counts and percentages. Statistical analyses were performed using IBM SPSS Statistics for Windows, Version 31 (IBM Corp., Armonk, New York, United States).

Due to the small sample size (n = 12), absence of a comparator group, and the descriptive, single-arm observational design, inferential statistics such as p-values, confidence intervals, or hypothesis testing were not applied. The study was not powered to detect statistically significant differences, and all analyses aimed to summarize feasibility, safety, and workflow characteristics during early adoption of robotic-assisted emergency surgery.

## Results

Population and preoperative details

A total of 12 consecutive patients were included in the study, of whom one (8.3%) was female. The mean age was 68.7 ± 18.0 years (median 73, range 38-91), and the mean body mass index (BMI) was 27.8 ± 2.6 kg/m² (median 27, range 25-34). Six patients (50.0%) had undergone prior abdominal surgery. The most common indication for emergency surgery was obstructing colorectal tumor (n = 7, 58.3%), followed by incarcerated hernia (n = 2, 16.7%), acute diverticulitis with obstruction (n = 1, 8.3%), intraoperative ureteric injury repair (n = 1, 8.3%), and bleeding colorectal tumor (n = 1, 8.3%) (Table 1).

**Table 1:** Patient demographics and preoperative characteristics. “Previous surgery” refers to any prior abdominal operation. BMI: body mass index

No.	Sex	Age (years)	BMI (kg/m²)	Previous surgery	Diagnosis – surgical indication
1	F	91	28	Yes	Obstructing transverse colon tumor
2	M	38	26	No	Obstructing upper rectal tumor
3	M	55	29.3	Yes	Obstructing sigmoid colon tumor
4	M	45	34	No	Acute diverticulitis
5	M	81	28	No	Obstructing cecal tumor
6	M	64	26	No	Ureteric injury
7	M	82	27	Yes	Incarcerated epigastric hernia
8	M	88	27	Yes	Obstructing ascending colon tumor
9	M	74	30	Yes	Obstructing rectal tumor
10	M	81	25	Yes	Bleeding ascending colon tumor
11	M	57	27	No	Incarcerated left inguinal hernia
12	M	72	26	Yes	Perforated diverticulitis

Intraoperative and postoperative outcomes

Colectomies were the most frequent procedure (n = 7, 58.3%), followed by hernia repairs (n = 2, 16.7%), anterior resections (n = 2, 16.7%), and ureteric repair (n = 1, 8.3%). The mean operative time was 3.5 ± 1.1 hours (median 3.0, range 2-6). The upper range reflected complex multisegmental resections (e.g., right colectomy with sigmoidectomy and extensive adhesiolysis for recurrent liposarcoma) and challenging pelvic dissections in obstructed rectal cancer. For standard right or left colectomies, OTs were within two to four hours, comparable to published emergency laparoscopic colectomy series [7,22]. No cases required conversion to open or laparoscopic surgery. Intraoperative drains were placed in five patients (41.7%) (Table 2).

**Table 2:** Surgical procedures and operative details. “Conversion” refers to conversion from robotic to open surgery. OT: operative time; TAPP: transabdominal preperitoneal

No.	Robotic-assisted emergency procedure	OT (hours)	Drain	Conversion
1	Transverse colectomy	3	No	No
2	Anterior resection	3	No	No
3	Right colectomy, sigmoidectomy, extensive adhesiolysis, and resection of recurrent liposarcoma tumours	6	Yes	No
4	Anterior resection	4	Yes	No
5	Anterior resection	4	No	No
6	Ureteric injury repair	3	Yes	No
7	TAPP hernia repair	2	No	No
8	Right hemicolectomy	3	Yes	No
9	Anterior resection and ileostomy	4	Yes	No
10	Right hemicolectomy and ileostomy	4	No	No
11	TAPP hernia repair	2	No	No
12	Hartmann’s procedure and intraabdominal abscess drainage	3	No	No

Complications and ICU admissions

No patient required ICU admission. Two patients (16.7%) experienced minor postoperative complications (Clavien-Dindo Grade I-II): The first patient presented a superficial surgical site infection after multisegmental colectomy, which was treated with intravenous antibiotics. The second case concerned an intraoperative ureteric injury during anterior resection, which was repaired robotically during the same operation without further sequelae. No major complications (Grade III-V) occurred. There were no reoperations, 90-day readmissions, or 90-day mortality (Table 3).

**Table 3:** Postoperative complications, management strategies, and ICU requirements. Only one patient experienced a complication (SSI), which was classified as grade I and treated with intravenous antibiotics. CEF: cefuroxime; ICU: intensive care unit; MET: metronidazole; SSI: surgical site infection.

No.	ICU	Complications	Clavien-Dindo grade	Management
1	No	None	–	–
2	No	None	–	–
3	No	SSI	I	CFX + MET
4	No	None	–	–
5	No	None	–	–
6	No	None	–	–
7	No	None	–	–
8	No	None	–	–
9	No	None	–	–
10	No	None	–	–
11	No	None	–	–
12	No	None	–	–

Postoperative recovery

All patients were mobilized on postoperative day one. The mean time to first flatus was 1.7 ± 0.6 days (median 2, range 1-2), and the mean time to first oral intake was 1.9 ± 0.5 days (median 2, range 1-3). Mean length of hospital stay was 3.7 (median 2, range 1-11), with prolonged stays observed only in patients undergoing extensive multisegment resections or requiring stoma formation. LOS in this series compares favorably with published emergency open colectomy data (median 7-10 days) and is similar to high-volume laparoscopic series [23,24] (Table 4).

**Table 4:** Postoperative data and outcomes. LOS: length of stay.

No.	Time to first flatus (days)	Time to mobilization (days)	Time to first oral feeding (days)	LOS (days)	90-day mortality	90-day readmission
1	1	1	1	2	No	No
2	2	1	2	2	No	No
3	2	1	2	11	No	No
4	2	1	2	2	No	No
5	2	1	2	2	No	No
6	2	1	2	11	No	No
7	1	1	1	1	No	No
8	2	1	2	4	No	No
9	3	1	3	6	No	No
10	1	1	2	2	No	No
11	1	1	2	2	No	No
12	2	1	1	2	No	No

## Discussion

This prospective single-center case series demonstrates that robotic-assisted surgery can be safely and effectively applied in selected emergency general surgical cases, with no conversions, major complications, readmissions, or mortality. Recovery milestones, including early mobilization and oral intake, were achieved promptly, and the median length of stay was two days, shorter than most reported open emergency colectomy series [7,22]. While the small sample precludes definitive conclusions, these findings add to the limited literature supporting the role of robotics in acute care surgery.

Open surgery remains the predominant emergency approach worldwide due to its speed and availability, but carries higher morbidity, wound complications, and prolonged hospitalization [8]. Laparoscopy offers reduced postoperative pain and faster recovery [24] but is often limited by rigid instrumentation, reduced maneuverability in confined spaces, and challenges in patients with bowel distension or dense adhesions [9,10].

Robotic platforms provide enhanced 3D visualization, wristed instruments with seven degrees of freedom, tremor filtration, and improved ergonomics. These advantages may be particularly relevant in emergencies involving distorted anatomy, pelvic pathology, or the need for precise suturing [12,13]. Docking time is often cited as a barrier; however, prior studies and our own experience show that in trained teams, set-up can be completed in under 10 minutes, similar to laparoscopy [14,15].

Comparative data are scarce. A recent World Society of Emergency Surgery (WSES) position paper [12] notes that robotic colectomy for obstruction has perioperative outcomes comparable to laparoscopy, with potentially lower conversion rates in complex scenarios. In our series, no conversions occurred, whereas conversion rates for laparoscopic emergency colectomy in the literature range from 10-25% [11,13].

Although no conversions occurred in our series, this finding should not be overinterpreted as evidence of platform superiority. Rather, it highlights the potential of robotics to enable minimally invasive completion of cases that, in our own experience, would likely have required conversion if attempted laparoscopically. These observations are hypothesis-generating and underscore the need for prospective comparative studies.

Emergency colorectal cases often present with massively distended bowel, making trocar placement and exposure challenging. In our protocol, controlled decompression via rectal tube, small enterotomy, or careful stepwise trocar insertion under direct vision was used to create a safe working space before docking. These measures, combined with articulated robotic instruments, facilitated dissection in restricted fields without conversion.

In incarcerated hernia repairs, the precision of robotic suturing and mesh placement was advantageous in friable or edematous tissue, allowing meticulous handling and tension-free repair. For intraoperative iatrogenic injuries, such as ureteric transection, the platform’s stability and magnification enabled immediate repair without conversion, an advantage rarely reported in emergency laparoscopic literature.

Successful robotic emergency surgery depends heavily on patient selection and team expertise. In this study, all cases were performed by a high-volume robotic colorectal and general surgeon (>250 cases) supported by a trained team with extensive robotic experience. The WSES consensus emphasizes that emergency robotic cases should be undertaken only by surgeons who have completed formal training and have substantial elective robotic experience [12].

In our setting, 24/7 access to the robotic platform eliminated selection bias related to equipment availability-a common limitation in other reports.

Limitations

This study has several limitations. The primary limitation is the very small sample size (n=12) and single-center, single-surgeon design, which inherently restricts statistical power and generalizability. Although all consecutive eligible emergency cases were included, the results reflect performance within an experienced, high-volume robotic unit and may not be directly applicable to lower-volume or less specialized settings. The absence of a comparator group prevents attribution of outcomes such as low conversion rates or short hospital stays solely to the robotic platform. Furthermore, operative time variability could not be formally compared with other approaches, and cost-effectiveness, resource allocation, and training requirements were not assessed. These limitations underscore the need for larger, multicenter, controlled studies to validate and expand upon our findings.

Future directions

To better define the role of robotics in acute care surgery, future research should focus on multi-center prospective studies including surgeons of varying experience levels and comparative analyses with laparoscopic and open approaches for matched indications. Procedure-specific protocols for emergencies (e.g., obstruction, strangulated hernia, perforation) and cost-benefit and resource utilization analyses in different healthcare settings are also necessary, while training pathway development for emergency robotic surgery, incorporating simulation and team-based drills, is another crucial step.

Interpretation and clinical relevance

Our findings suggest that, when performed by a trained team with continuous platform access, robotic-assisted surgery can be a feasible option for selected emergency cases. However, its use should not be generalized to all acute presentations and must be weighed against patient condition, surgical expertise, and institutional resources. In high-risk patients, the potential benefits of minimally invasive access, precise dissection, and rapid recovery may justify its use, but further evidence is needed to guide widespread adoption.

## Conclusions

This case series suggests that robotic-assisted surgery may be a safe and feasible option for selected emergency general surgical procedures when performed by experienced surgeons within a trained, coordinated team and in an institution with continuous robotic platform availability. In our cohort, no conversions, major complications, readmissions, or mortality were observed, and postoperative recovery was favorable. The potential advantages of robotics in emergencies may be particularly relevant in complex pelvic dissections, obstructing colorectal tumors, and incarcerated hernias, where precise dissection, ergonomic suturing, and enhanced visualization are critical. Nonetheless, these findings must be interpreted with caution, given the small sample size, single-center design, absence of a control group, and reliance on a highly experienced team.

Robotic-assisted surgery in emergencies should not be regarded as a universal substitute for open or laparoscopic approaches, but rather as an additional tool in the acute care surgeon’s armamentarium. Its use should be reserved for carefully selected patients and supported by appropriate institutional infrastructure and training. Notably, in this small series, all cases were completed robotically without conversion, outcomes that, in our own experience, would have been highly likely to require conversion if performed laparoscopically. This suggests a potential advantage of robotics in selected high-difficulty scenarios, although our findings remain hypothesis-generating and require validation in larger, controlled studies. Future research should therefore focus on multicenter, comparative designs incorporating cost-effectiveness analyses to better define patient selection, refine protocols, and evaluate outcomes across diverse healthcare settings and levels of experience.
